# AI-Based Prediction of Post-ERCP Pancreatitis: A Comparative Study Using Tabular, Image, and Multimodal Data

**DOI:** 10.3390/diagnostics16121824

**Published:** 2026-06-12

**Authors:** Anum Jamil, Waseemullah Nazir, Abeer Altaf, Saad Khalid Niaz

**Affiliations:** 1 Department of Computer Science and Information Technology, NED University of Engineering and Technology, Main University Road, Karachi 75270, Sindh, Pakistan; jamil.pg4001025@cloud.neduet.edu.pk; 2 Sindh Institute of Advance Endoscopy and Gastroenterology, Heritage Building 1st Floor, Dr Ruth K.M. Pfau Civil Hospital, Baba-e-Urdu Road, Karachi 74400, Sindh, Pakistan; abeeraltaf.98@gmail.com (A.A.); saadniaz@yahoo.co.uk (S.K.N.)

**Keywords:** post-ERCP pancreatitis, PEP prediction, endoscopic image analysis, data augmentation, class imbalance, multimodal fusion

## Abstract

**Background/Objectives**: Post-Endoscopic Retrograde Cholangiopancreatography Pancreatitis (PEP) is a clinically significant complication of ERCP, occurring in approximately 2–10% of general cases and at higher rates in high-risk patients. Early prediction of PEP risk may support timely intervention and improved patient management. This retrospective single-center study comparatively evaluated tabular clinical data, endoscopic image data, and multimodal fusion approaches for PEP prediction. **Methods**: Retrospective data collected from the Sindh Institute of Advanced Endoscopy and Gastroenterology were analyzed using machine learning and deep learning techniques. XGBoost(version 3.2.0) was applied to tabular clinical data, while EfficientNet-B0, ResNet50, and DenseNet201 were used for endoscopic image analysis. A multimodal contrastive learning (MMCL)-based framework combining ResNet50 image features with multilayer perceptron (MLP)-based tabular features was additionally implemented for binary PEP prediction. Class imbalance mitigation techniques, including data augmentation and balancing strategies, were applied during training. Model performance was evaluated using the area under the receiver operating characteristic (ROC) curve (AUC), sensitivity, F1-score, and precision. SHAP analysis was performed to identify important predictive features. **Results**: The tabular XGBoost model achieved the best predictive performance with an AUC of 0.95 and a sensitivity of 0.50, while five-fold cross-validation yielded an AUC of 0.79 and a sensitivity of 0.48. Among image-based models, ResNet50 achieved the highest performance, with an AUC of 0.76 and a sensitivity of 0.40. The multimodal model achieved an AUC of 0.57 and a sensitivity of 0.20. SHAP analysis identified cannulation time, ampulla type, and age as prominent features associated with PEP prediction. **Conclusions**: This exploratory study suggests that structured clinical data currently provide stronger predictive signals for PEP prediction than the available image and multimodal data within this limited cohort. The relatively low occurrence of PEP contributed to class imbalance despite mitigation strategies. Future multicenter studies with larger datasets, improved image availability, synthetic data generation, and advanced multimodal fusion techniques may improve predictive performance and clinical applicability.

## 1. Introduction

The application of artificial intelligence (AI) in diagnostic and prognostic processes is becoming essential. This is particularly true for the prediction of Post-Endoscopic Retrograde Cholangiopancreatography (ERCP) pancreatitis (PEP) [1,2]. PEP is a common complication of the ERCP procedure and can be damaging for patients [3]. The ability to accurately predict PEP would be beneficial for reducing patient suffering and improving clinical decision-making and treatment [4,5].

With the recent emergence of sophisticated machine learning methods and sufficient amounts of structured clinical data and high-quality endoscopic data, this research aims to establish predictive models for early identification of high-risk subjects. In risk classification processes, clinical judgment and retrospective approaches have been the benchmarks. However, modern risk classification processes shift the focus to clinically relevant data. In endoscopic imaging [6], most temporally and spatially sensitive data layers contain difficulty and inflammation risk indicators, particularly in the images of the ampulla of Vater. These data layers are often fully accessible to deep convoluted models like EfficientNet. On the other hand, data layers pertaining to patient demographics (e.g., age, sex) and clinical history (e.g., co-morbidities, labs) constitute central data points in the risk classification processes.

This research comparatively evaluates tabular clinical data, endoscopic image data, and multimodal fusion approaches for the prediction of post-ERCP pancreatitis (PEP). Given the limited availability of positive PEP cases and the dataset’s single-center, retrospective nature, this work was designed as an exploratory proof-of-concept study to evaluate the feasibility of AI-based PEP prediction using clinical and endoscopic data. Such approaches may contribute to the future development of intelligent decision-support systems for gastroenterologists to streamline risk assessment, enhance procedural safety, and improve patient care.

### Literature Review

The literature covers attempts to forecast complications following ERCP, the contribution of image analysis to PEP, and the proposed benefits of combining multiple techniques to improve diagnostic precision. Here, the intention is to critically evaluate these approaches, determine their strengths and weaknesses, and identify the basis for the techniques used in the current research. Accordingly, the analysis focuses on three key components: predictive analytics using tabular data, analysis of images related to ampulla morphology [7,8], and the use of multimodal deep learning for medical image analysis [9,10].

In the past, risk assessment relied solely on clinician prediction and retrospective studies, which prevented modern evidence-based techniques from using clinically relevant data to improve diagnostic and prognostic workflows [3]. In endoscopic imaging, the presence of temporally and spatially distinct layers can create an image that indicates difficulty (and an increased risk of inflammation), particularly in situations involving the ampulla of Vater. These critical imaging attributes can be captured using deep convolutional models such as EfficientNet [11]. Structured clinical data [4,5,12,13,14,15] involving patient age, sex, comorbidities, and laboratory values (i.e., as a single entity) remains the most relevant data for risk assessments and stratification.

Each of these methods has been the focus of research in isolation. Models that effectively stratify risk post-ERCP using structured clinical data, like tree-based ensembles and gradient boosting, have been demonstrated in the literature [4,5,12,13,14,15]. While clinical risk stratification models are interpretable and rely on readily accessible clinical data, they do not account for the clinical anatomy depicted in endoscopic images. In contrast, clinical anatomy models using image-based data rely on CNNs to assess ampulla anatomy and estimate the difficulty of the procedure [4,7]. These models suggest that the presence of certain ampulla types (particularly Type 3 and 4) can increase the difficulty of cannulation and the risk of post-ERCP pancreatitis (PEP). However, clinical risk stratification models based on images often lack patient-level clinical data, limiting their predictive usefulness.

The combination of image and clinical data using multimodal methods has the potential to address the aforementioned missing data. A case in point is the application of contrastive learning to the clinical and imaging data of the same patient, resulting in the creation of a shared conceptualized predictive space [9]. Attention-based fusion modules can also integrate 3D imaging data with corresponding clinical data [10]. Despite these methods showing the potential of multimodal learning, they can be computationally intensive and are often designed for non-2D imaging data, making them less applicable to endoscopic ERCP images.

Despite the growing body of literature, significant gaps remain. Very few studies have addressed ampulla image analysis integrated with clinical attributes for predicting PEP. Additionally, the existing multimodal frameworks are excessively complicated for direct clinical applications. This study begins to fill these gaps by using an ERCP dataset to assess the practical utility of multimodal learning for predicting PEP. We use a clinically relevant fusion mechanism to integrate ampulla morphology from endoscopic images with the patients’ clinical variables. The study goes beyond predictive performance and provides a differentiation analysis of the image and clinical features, thereby aiding the gastroenterologists in their risk assessment and procedural planning.

In the area of ERCP PEP prediction, this research aims to:

1. Create a multimodal artificial intelligence framework using 2D images of the ampulla and clinical data to integrate PEP prediction.
2. Analyze the imaging and clinical data, both independently and in conjunction, for their predictive contributions.
3. Improve the safety of the procedure and the quality of the care provided to patients by providing clinically relevant insights.

In Gastroenterology, this research provides the first step toward developing a practical decision-making support tool to help predict PEP risk and close a significant gap in the use of Artificial Intelligence.

## 2. Methodology

The methodology for this exploratory proof-of-concept study on post-endoscopic retrograde cholangiopancreatography (ERCP) pancreatitis (PEP) prediction was implemented in Python version 3.12.13 using Google Colab. The following software libraries were used: PyTorch version 2.10.0+cu128, TorchVision version 0.25.0+cu128, Matplotlib version 3.10.0, scikit-learn version 1.6.1, and XGBoost version 3.2.0. Computations were performed using NVIDIA CUDA-enabled GPU acceleration.

**Figure 1:**
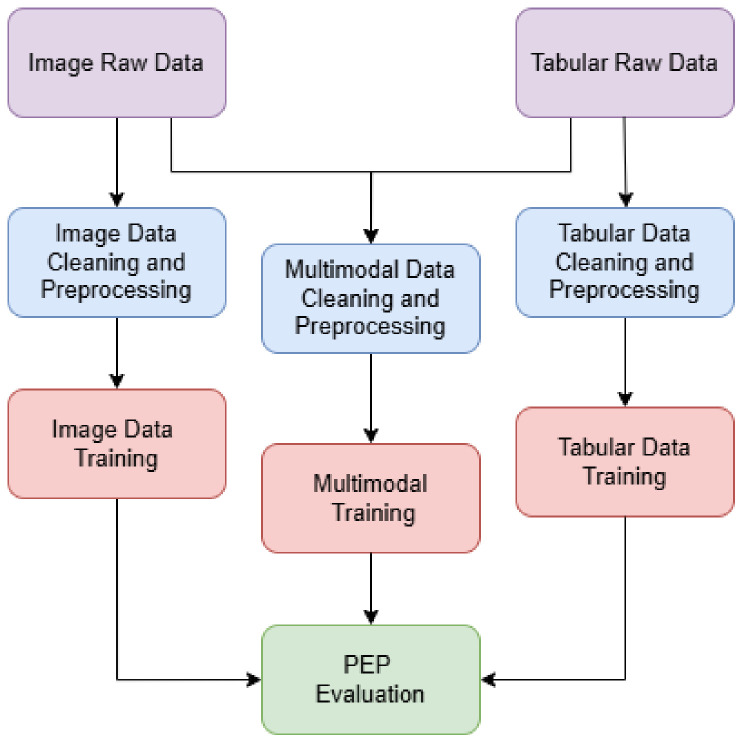
Methodology Diagram.

The study integrates both tabular and image data to enable comparative multimodal analysis (Figure 1). It covers data preprocessing, model architectures for tabular and image modalities, implementation of a multimodal fusion approach, and evaluation techniques. Given the retrospective single-center dataset and limited PEP cases, the study evaluated AI-based PEP prediction using clinical and endoscopic data.

### 2.1. System Architecture

Data captured from two different modalities (tabular data + endoscopic images) is designed in the system architecture to acquire more advanced information. Each data domain analyzes its specific modality using a dedicated model, and a fusion model integrates the latent components of the two to enhance the robustness of the prediction.

### 2.2. Classification of Tabular Data Using XGBoost

In a risk assessment of post-ERCP pancreatitis (PEP) encounters, the importance of structured data in clinical practice cannot be emphasized enough. In this analysis, tabular features, including PEP occurrence, patient demographics, scores for cannulation difficulty, procedure time, and the techniques used, were found to be highly correlated with PEP occurrence.

#### Training and Evaluation

The dataset was split into 80% for training and 20% for testing. To handle class imbalance, the majority class was randomly downsampled in the training data. A 5-fold cross-validation was conducted on the training set for hyperparameter tuning and performance stability assessment.

### 2.3. Image-Based Classification Using EfficientNet-B0, DenseNet-201, and ResNet-50

The shape, dimensions, and texture of the ampulla of Vater [16] are predictive of PEP. For this study, the classification of medical images used deep convolutional neural networks (CNNs) [17,18,19,20,21,22,23,24,25,26], which have demonstrated great success in this domain. In order to evaluate a variety of model architectures with respect to depth, parameters vs. performance, and the ability to learn features effectively, three models were chosen:

**EfficientNet-B0** [11] for the best parameter-to-performance ratio.

**DenseNet-201** [27] for highly connected and feature-reuse dense networks.

**ResNet-50** [11] for a strong deep residual network capable of deep representation learning.

This approach allows us to assess each model’s effectiveness in classifying post-ERCP pancreatitis based on ampullary morphology.

#### 2.3.1. Data Augmentation and Class Balancing

To improve the model’s robustness and reduce overfitting, data augmentation was applied solely to the training sets. Transformations were applied per modality (e.g., intensity-based modifications for images). To address class imbalance, the majority class was randomly downsampled, and class weighting was applied during training. This resulted in a more balanced training distribution and improved generalization.

#### 2.3.2. Model Architectures

**Figure 2:**
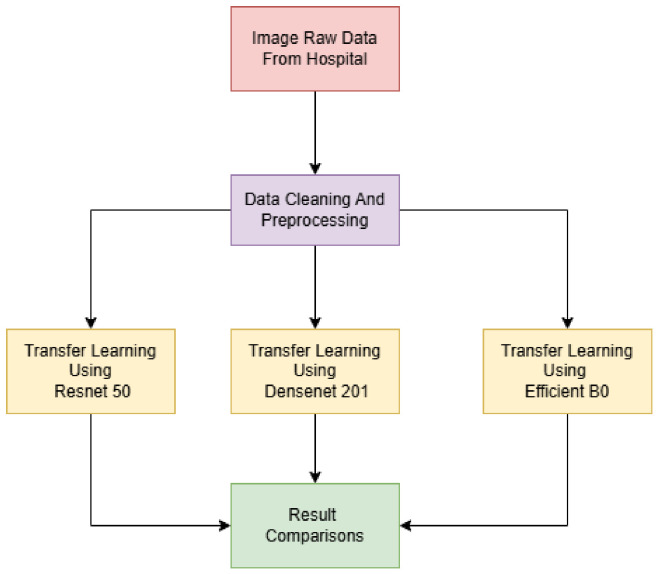
Image Learning Flow.

Three different Transfer learning-based Convolutional Neural networks were used due to their lighter weight and high performance (Figure 2).

EfficientNet-B0 was constructed using a compound scaling technique where network depth, width, and resolution are uniformly scaled using a fixed coefficient. Compared to other models with the same accuracy, EfficientNet-B0 is more lightweight and more efficient, with better representational power. For this study, the classification head was modified to include a binary softmax layer to classify PEP and non-PEP outcomes.

DenseNet-201 is composed of densely connected convolutional blocks, where all prior layers are inputs, encouraging the reuse of features and improving gradient flow. This unique pattern of connected layers enables the network to identify minute details of the visual image, particularly the intricate anatomical features of an endoscopic image. The final layer was altered to produce two classes, indicating whether PEP was present.

ResNet-50 is a deep residual network that uses an identity shortcut connection, allowing deeper architectures to be more easily trained by alleviating the vanishing gradient problem. These residual blocks enable the more rapid and efficient development of layered features. The model’s fully connected layer was modified to produce a binary PEP prediction.

#### 2.3.3. Training and Evaluation

All models were retrained using specific transfer learning techniques. At the start, the feature extractor layers were frozen, and training was only performed on the newly added classifier head. Subsequently, deeper layers were unfrozen in stages to adapt the feature representations to the medical domain.

The models were developed using cross-entropy loss and either Adam or SGD optimizers. A stratified train–test split (80/20) was used to preserve class distribution, and no separate validation set was created for image-based experiments due to the limited number of positive cases.

### 2.4. Multimodal Fusion Using Multimodal Contrastive Learning (MMCL) [9]

To jointly leverage anatomical image information and structured clinical variables, a multimodal contrastive learning (MMCL)-based framework [9] was implemented. The model combined a ResNet50 image encoder with a multilayer perceptron (MLP)-based tabular encoder. The framework consisted of self-supervised multimodal pretraining followed by supervised fine-tuning for binary PEP prediction.

#### 2.4.1. Self-Supervised Pretraining Phase

The multimodal framework utilized a SimCLR-inspired contrastive learning strategy to align image and tabular representations within a shared latent space.

1. **Image Encoder**: A ResNet50 backbone pretrained on ImageNet was used to extract image features. The final fully connected layer was removed, producing 2048-dimensional embeddings from endoscopic images resized to 128 × 128 pixels.
2. **Tabular Encoder**: Structured clinical variables (28 features) were processed using a two-layer MLP-based encoder with batch normalization and ReLU activation, producing 2048-dimensional embeddings.
3. **Projection Heads**: Separate SimCLR projection heads were applied to both modalities, each consisting of a linear layer, ReLU activation, and a final linear layer mapping embeddings to a 128-dimensional latent space.
4. **Contrastive Learning Objective**: A contrastive loss was used to maximize similarity between matched image–tabular pairs from the same patient while minimizing similarity between unmatched pairs.

#### 2.4.2. Supervised Fine-Tuning

After pretraining, projection heads were removed and encoders were fine-tuned for PEP classification.

1. **Feature Fusion**: Image and tabular embeddings (2048 dimensions each) were concatenated to form a 4096-dimensional fused feature vector.
2. **Classification Head**: The fused representation was passed through a fully connected layer for binary classification.
3. **Loss Function**: Cross-entropy loss was used for optimization.
4. **Optimization**: Training was performed using the Adam optimizer with learning-rate scheduling.

#### 2.4.3. Multimodal Training and Evaluation

For the multimodal studies, the dataset was divided into 60% for training and 20% each for validation and testing. Modality-specific preprocessing and data augmentation were used only on the training set. Endoscopic images were resized to 128 × 128 pixels prior to model input. To improve model fairness, the majority class was downsampled in the training set to address class imbalance.

### 2.5. Outcome Labeling Strategy

Labeling was performed based on the post-procedure follow-up. Any patient who met clinical criteria for PEP during follow-up was labeled positive, regardless of severity.

## 3. Dataset Description

This provides an in-depth account of the dataset for the study, which aims to predict post-endoscopic retrograde cholangiopancreatography pancreatitis (PEP) using tabular clinical data, endoscopic images of the ampulla of Vater, and a multimodal fusion of these data types. The dataset consists of structured and image data and was created in partnership with the Sindh Institute of Advanced Endoscopy and Gastroenterology (SIAG) at Civil Hospital Karachi. The dataset’s high dimensionality and heterogeneity will enable the construction of robust machine learning and deep learning models.

### 3.1. Time Period

The study used retrospectively collected clinical and endoscopic data from May 2023 to April 2025 at the Sindh Institute of Advanced Endoscopy and Gastroenterology (SIAG), Karachi. The data had been prospectively recorded during routine clinical practice and included endoscopic image acquisition, procedural and clinical information, post-procedure follow-up, and outcome validation.

### 3.2. Inclusion and Exclusion Criteria

#### 3.2.1. Inclusion Criteria

1. Patients undergoing ERCP at SIAG with available ampulla images.
2. Availability of clinical and procedural data for structured (tabular) analysis.
3. Patients with complete follow-up records confirming or ruling out PEP.

#### 3.2.2. Exclusion Criteria

1. Patients who have ampulla images that are of poor quality or images that are missing.
2. Clinical data that is incomplete or missing.
3. Patients who have a history of pancreatic disease that may confound the diagnosis of PEP.
4. Patients who have a history of ERCP.

### 3.3. Data Composition and Statistics

The dataset comprises two primary components:

#### 3.3.1. Tabular Data

**Figure 3:**
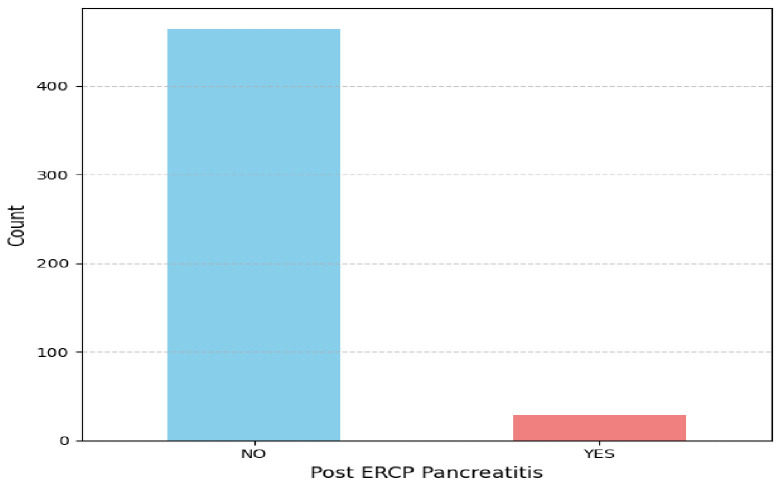
PEP Distribution.

As per the procedural logs and the medical records of the patients, structured information was retrieved. The dataset in tabular form contains demographics, clinical findings pre- and post-procedure, and ERCP-specific technical variables. The features along the labels include the following:

*PEP (post-ERCP pancreatitis)*: PEP [28] was used as the binary outcome label indicating whether the patient developed post-ERCP pancreatitis (Yes/No). PEP was defined according to standard consensus criteria as the presence of new or worsening abdominal pain following ERCP associated with serum amylase or lipase levels elevated to at least three times the upper limit of normal approximately 24 h after the procedure, requiring hospitalization or prolongation of planned admission. Data analysis (Figure 3) showed that the dataset was highly imbalanced, with relatively few positive PEP cases due to the low incidence of the condition.

Initially, the distribution of PEP cases was: No: 464 and Yes: 29.

After data cleaning, the distribution is:

No: 419 and Yes: 27.

**Figure 4:**
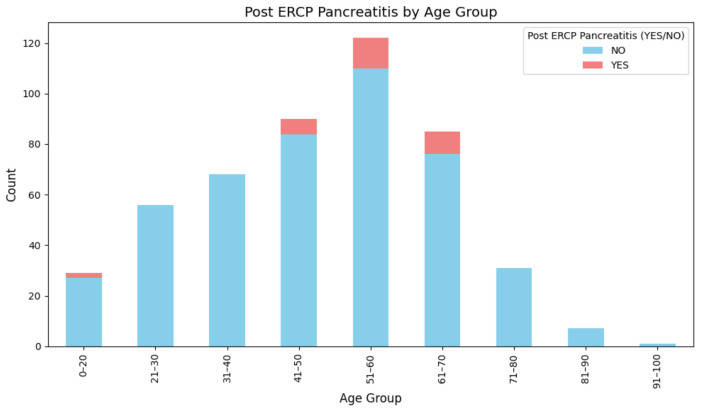
Age Distribution with respect to PEP.

*Age*: Integer value representing the patient’s age. After data analysis, it was found that patients aged 40–70 are more likely to have PEP. The age distribution is shown in Figure 4.

**Figure 5:**
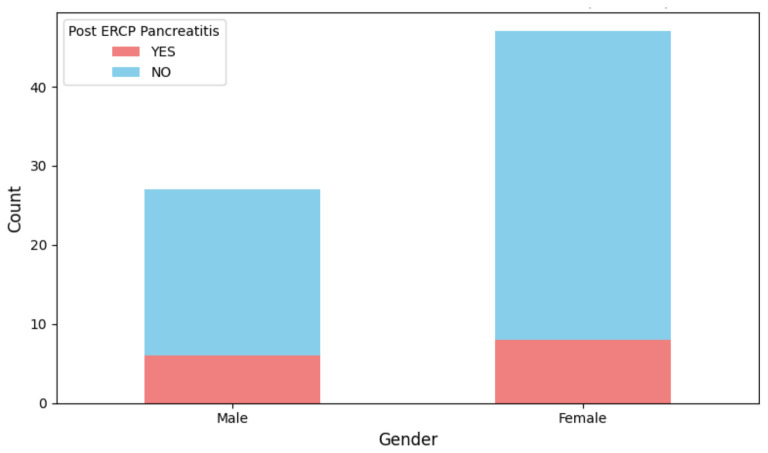
Gender Distribution with respect to PEP.

*Gender*: categorical variable (Male/Female). Analysis shows that females have a higher chance of developing PEP (Figure 5).

**Figure 6:**
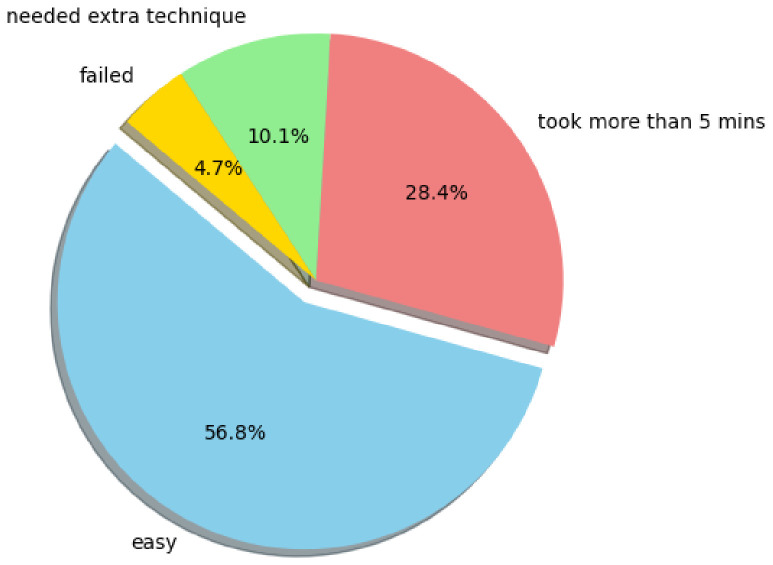
Proportion of Cannulation Difficulty Level.

*Cannulation Difficulty Level*: Categorical variable (Figure 6) indicating technical complexity, categorized as:

1. Easy,
2. Needed Extra Technique,
3. Took More Than 5 min,
4. Failed.

*Cannulation (biliary)*: Success or failure of biliary cannulation (Yes/No).

*Sphincterotomy*: Whether a sphincterotomy was performed (Yes/No).

*Sphincteroplasty*: Whether sphincteroplasty was performed (Yes/No).

**Figure 7:**
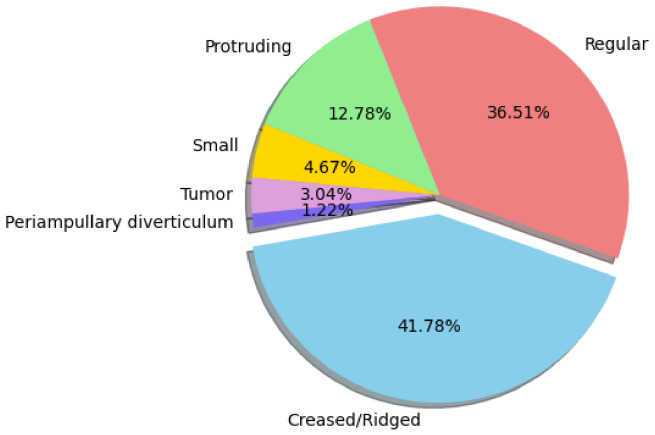
Proportion of Ampulla Type.

Ampulla Type: Morphological classification was initially based on four types as described by Haraldsson et al. [8,29]. However, Type 5 and Type 6 could not be categorized into the original four types, so they were added as separate classes. The final classification includes six types, as shown in Figure 7.

Type 1: Regular,

Type 2: Small,

Type 3: Protruding,

Type 4: Creased/Ridged,

Type 5: Tumorous (separate class for ampulla with visible tumors),

Type 6: Periampullary diverticulum.

*No of attempts*: Number of cannulation attempts (categorical).

*Cannulation time*: Time taken for cannulation in minutes (float).

*Bilirubin*: The patient’s bilirubin level, typically measured in mg/dL (float).

*Jaundice*: Indicates the presence of jaundice in the patient, commonly recorded as a binary feature (Yes/No).

*Abdominal Pain*: Indicates whether the patient reported abdominal pain prior to the procedure (Yes/No).

*Stone Removal*: Whether stone removal was performed (Yes/No).

*Special Technique Used*: Encoded as:

0 = No special technique,

1 = Special technique used.

The tabular features include both pre-procedural clinical variables (e.g., age, gender, bilirubin, jaundice, abdominal pain, and ampulla type) and intraprocedural parameters (e.g., cannulation time, number of attempts, cannulation difficulty level, sphincterotomy, and related procedural interventions). As the outcome of interest is post-Endoscopic Retrograde Cholangiopancreatography (ERCP) pancreatitis (PEP), these variables are used to predict the likelihood of PEP occurrence through a binary classification model based on combined pre- and intraprocedural data.

#### 3.3.2. Image Data

High-resolution endoscopic images of the ampulla of Vater were collected during ERCP procedures. As we know, the dataset is imbalanced. Additionally, we do not have corresponding image records for all tabular entries because some images were not recorded, and some were unclear. As a result, the image dataset we obtained for PEP classification includes:

No = 360 images,

Yes = 24 images,

Total = 384 images.

Due to this imbalance, two different training strategies were applied:

Imbalanced Data: Augmentation was applied only to the ‘Yes’ class, with each image augmented 11 times to increase its representation in the training data.

Balanced Data: A subset of ‘No’ class cases was dropped to balance the dataset, resulting in: No = 24 images, Yes = 24 images.

#### 3.3.3. Multimodal Data

In this multimodal approach, both image and tabular data were utilized. The dataset size has been reduced due to the requirement that both data types be available for each instance. Additionally, since the data was highly imbalanced, a portion of the majority class (‘PEP: No’) was removed to improve class balance. Consequently, the distribution of other features has also been altered.

Initially, the data proportion was as follows:

No = 289 images,

Yes = 22 images.

Now it is:

No = 75 images,

Yes = 22 images.

### 3.4. Data Preprocessing

#### 3.4.1. Tabular Data

Missing value records were dropped. Categorical variables were label-encoded or one-hot-encoded depending on the model’s requirements. Continuous features such as age and cannulation time were normalized or standardized as needed.

#### 3.4.2. Image Data

**Figure 8:**
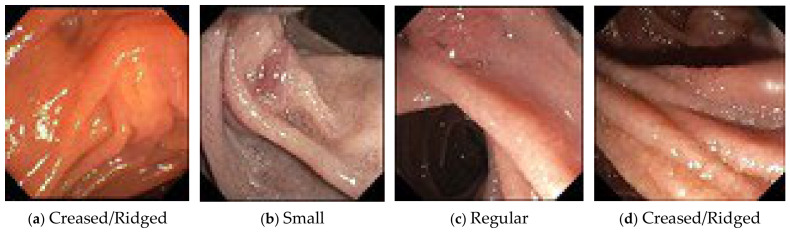
Ampulla Images before preprocessing.

**Figure 9:**
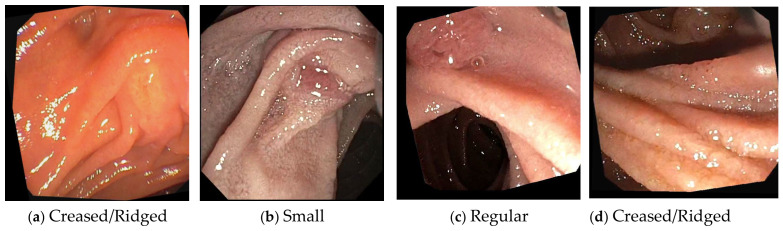
Ampulla Images after preprocessing.

Automatic image transformations were applied as part of the transfer learning preprocessing pipeline. To address class imbalance and increase the number of positive PEP samples during training, multiple data augmentation techniques were applied, including random rotations, horizontal and vertical flips, brightness normalization, zooming, and geometric transformations. Approximately 11 augmented variations were generated from each original positive-case image during training. Figure 8 and Figure 9 present four randomly selected example images labeled (a–d). Figure 8 shows the original endoscopic images before preprocessing and augmentation, while Figure 9 shows one corresponding augmented/preprocessed example generated from each original image. The labels (a–d) are used only to preserve correspondence between the original and augmented versions of the same cases and do not represent anatomical categories or clinical subtypes.

#### 3.4.3. Multimodal Data

The tabular data CSV was updated to include an image filename column, with the corresponding file name added to each record. This helps link the image data with the tabular data during training.

1. A total of 3 files were saved to be used as input. Each of these three types of files is further divided into train, test, and validation sets. One file contains the labels in .pt format.
2. Another file contains all tabular features.
3. Third files contain image paths in .pt format.

## 4. Results

The results were obtained from various machine learning and deep learning models applied to tabular data, endoscopic image data, and their multimodal fusion. The aim is to identify the best-performing approach for predicting post-ERCP pancreatitis (PEP). The results are analyzed using standard evaluation metrics: accuracy, precision, recall, F1-score, and a confusion matrix. Each modality is evaluated individually, followed by a comparison of performance across different modalities to determine whether a multimodal approach offers improved predictive power.

### 4.1. Evaluation Metrics

To ensure a performance assessment is valid and thorough, these metrics were selected.

- Accuracy: proportion of instances identified and classified correctly,
- Precision: positive cases identified correctly by the model,
- Recall (Sensitivity): the model’s ability to identify all of the actual positive instances,
- F1 Score: precision and recall’s harmonic mean,
- Confusion Matrix: method for performance of class-oriented prediction.

### 4.2. Result on Tabular Data

The XGBoost model was employed to train on the tabular data. Two training strategies were used:

1. Train–test split, with 80% of the data used for training and 20% for testing.
2. 5-fold cross-validation to ensure more reliable evaluation.

#### 4.2.1. Train and Test Split

**Figure 10:**
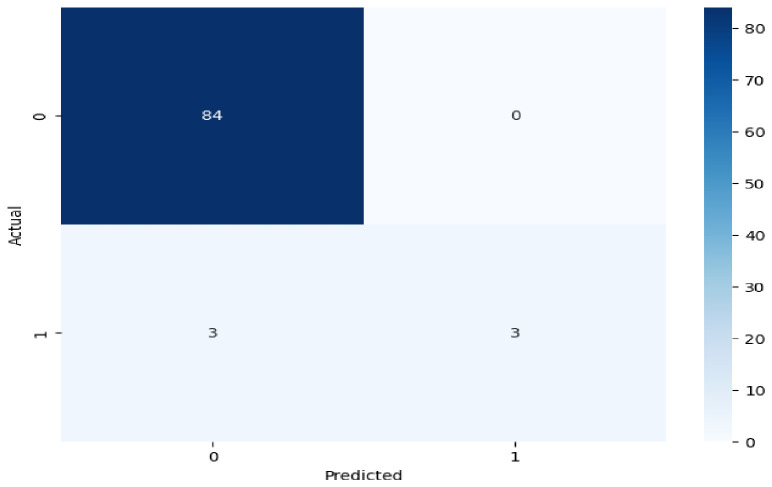
Tabular Train and Test Split Result.

Confusion Matrix and Classification Report:

The confusion matrix (Figure 10) and associated classification metrics (Table 1) were used to evaluate the model’s performance on the test dataset. To address class imbalance, the scale_pos_weight parameter was included during XGBoost training, enabling greater emphasis on the minority class (PEP: Yes). The model was trained using XGBoost on structured tabular data.

Class 0 (PEP: No) has 84 true positives and 0 false positives, resulting in a precision of 0.97, recall of 1.00, and F1-score of 0.98. Class 1 (PEP: Yes) has 3 true positives and 3 false negatives, yielding a precision of 1.00, recall of 0.50, and F1-score of 0.67. From the confusion matrix, it is clear that model performs very well in identifying the majority class (PEP: No). However, it struggles with the minority class (PEP: Yes), misclassifying 3 of 6 positive cases as negative.

**Figure 11:**
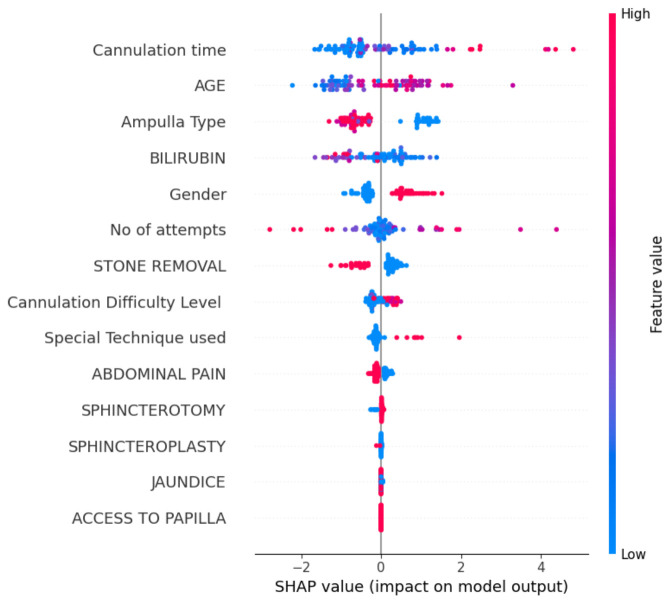
SHAP Analysis.

SHAP: The SHAP (SHapley Additive exPlanations) module was used to interpret the XGBoost model’s output. In the graph below (Figure 11), red dots represent high feature values, purple indicates moderate values, and blue signifies low values. The features listed along the y-axis (from top to bottom) are ranked by their importance for prediction. For example, Cannulation Time appears at the top, indicating it is the most influential predictor of PEP.

On the x-axis, the right side corresponds to predictions of ‘PEP: Yes’, and the left side to ‘PEP: No’. According to this analysis:

1. High Cannulation Time values are associated with a higher likelihood of PEP.
2. Age, when high to moderate, also contributes to a ‘Yes’ prediction.
3. Easy cannulation is linked with ‘Yes’ predictions.
4. Low bilirubin levels are associated with ‘Yes’ predictions.
5. Features listed near the bottom of the y-axis appear to have minimal impact on the prediction.

However, the model still needs improvement to understand all the features and their correct influences on PEP prediction.

#### 4.2.2. Cross Validation

**Figure 12:**
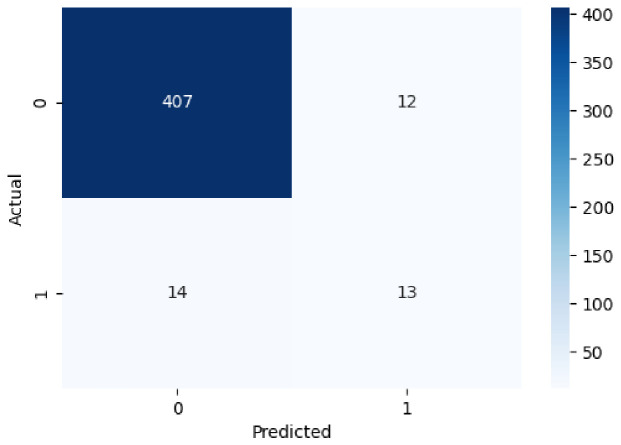
Tabular Cross Validation Result.

Confusion Matrix and Classification Report:

To assess the robustness of the XGBoost model on the tabular dataset, 5-fold cross-validation was performed. Figure 12 shows the confusion matrix for aggregated predictions across all folds.

An imbalance in the dataset is concerning: there are more PEP: No (Negative) cases than PEP: Yes (Positive) cases. Of the 446 samples, 419 were negative, and 27 were positive. Scaleposweight still helped the model identify 13 positive cases. Precision and recall were still lower for the positive class than for the negative class, but they improved after training without addressing the imbalance (Table 2).

**Figure 13:**
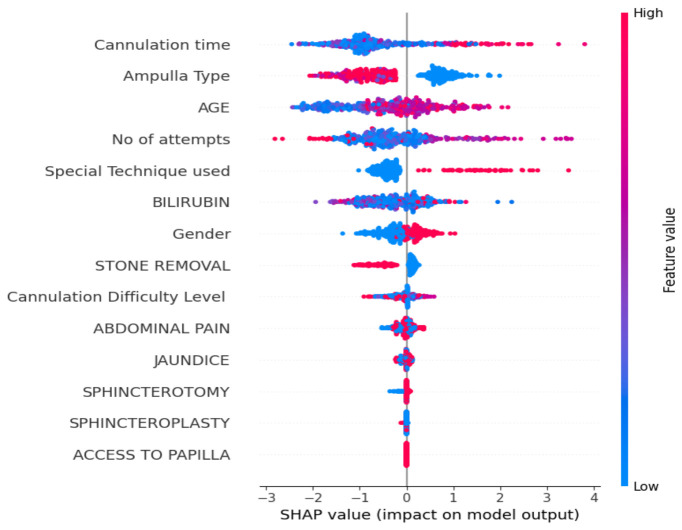
SHAP Summary on XGBoost CV Result.

SHAP:

Below is a SHAP (SHapley Additive exPlanations) plot detailing the features contributing to the XGBoost model’s predictions on Post-ERCP Pancreatitis (PEP). SHAP analysis (Figure 13) was employed to explain the XGBoost model’s features, and the provided SHAP summary plot shows that the most significant features for predicting Post-ERCP Pancreatitis (PEP) are Cannulation Time, Ampulla Type, and Age. Greater cannulation time and increased age are associated with a higher risk of PEP, as is the use of special techniques.

Features such as stone removal and jaundice are of comparatively lesser importance overall and fall within the realm of clinical expectations, aiding understanding of the model.

### 4.3. Results on Image Data

To address performance issues with the PEP classification models and data imbalance, two strategies were implemented. These strategies are as follows: Image Augmentation, creating more samples for the minority class (PEP), and Image Balancing, where the majority class is reduced to equal the minority class.

#### 4.3.1. Training and Testing Loss and Accuracy with Data Augmentation

**Figure 14:**
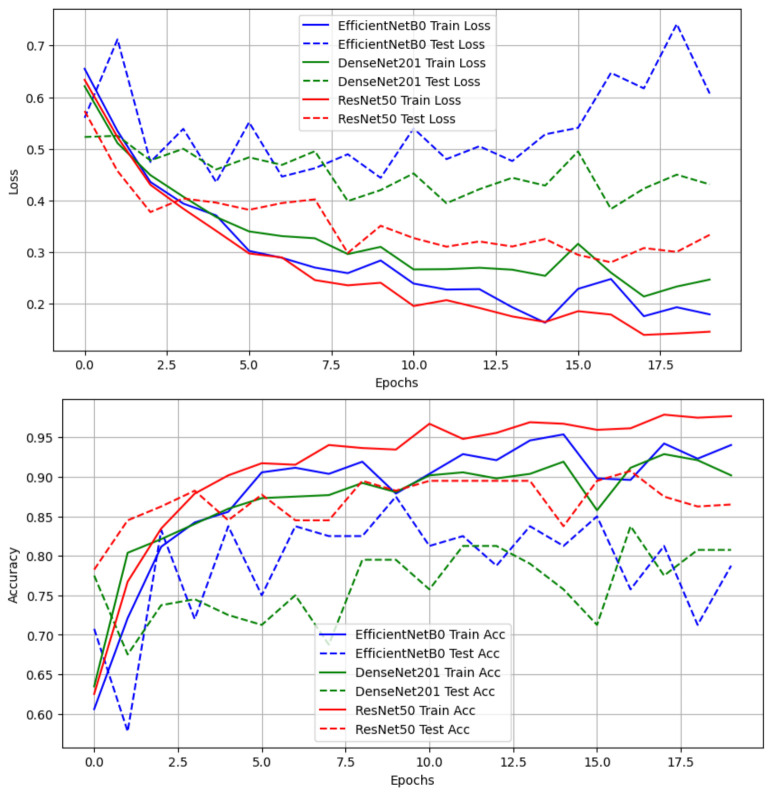
Training and Test Loss and Accuracy with Data Augmentation.

This figure (Figure 14) shows the performance of data augmentation on the training dataset of EfficientNetB0, DenseNet201, and ResNet50.

The figure shows the loss curves, and the bottom figure shows the accuracy plots over 20 epochs. With augmentation, ResNet50 had the best and most consistent performance, with a test loss that declined and a test accuracy that was high and stable. The training and test curves align, suggesting strong generalization. DenseNet201 also performed well, with high, stable test accuracy, suggesting it has reached a plateau and is not optimizing. EfficientNetB0 achieved high accuracy on the training data but was highly unstable in terms of test loss and accuracy, suggesting it might be sensitive to overfitting or to the use of augmented data. Overall, ResNet50 showed the most stable and consistent improvements with data augmentation.

While EfficientNetB0 seems to need more tuning or regularization to stabilize its performance across epochs, DenseNet201 shows reasonable generalization.

#### 4.3.2. Training and Testing Loss and Accuracy with Data Balancing

**Figure 15:**
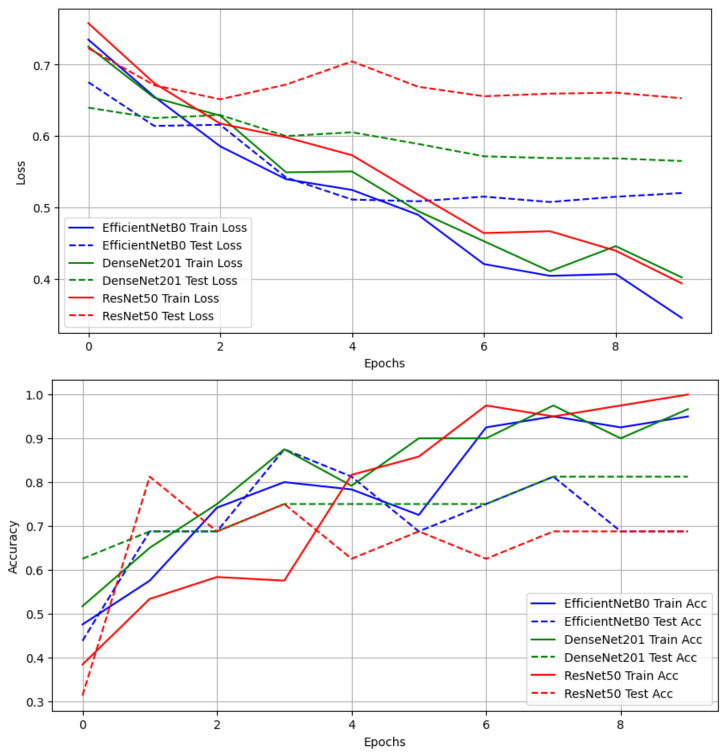
Training and Test Loss and Accuracy with Data Balancing.

This figure (Figure 15) shows the training and test results for EfficientNetB0, DenseNet201, and ResNet50, with data balancing achieved by removing instances of the majority class. The top figure shows the loss trends during testing, and the bottom figure shows the test accuracy over 10 epochs. Overall, after balancing, DenseNet201 achieves the best performance on final test loss and test accuracy, and shows a consistent, stable trend with no noticeable deviations. EfficientNetB0 shows high performance with a stable, smooth test curve and only a small dip during training.

Although the loss is slightly lower than that of DenseNet201, it exhibits high stability. In contrast, ResNet50 exhibits the lowest test accuracy and the highest test loss, resulting in a flat, ineffective test performance curve. Despite strong accuracy and loss metrics for DenseNet201, it needs further evaluation to focus on class-wise generalization. Above-average accuracy can be a sign of class-wise discrimination that places undue emphasis on certain classes, and understanding how precision, recall, and F1 scores are balanced is key. This class-wise performance and confusion matrix-based evaluation approach is essential for ensuring the model generalizes across classes and is even more crucial in medical imaging tasks that require detecting the minority class.

#### 4.3.3. Summarize Evaluation Results

Class balancing improved the model’s generalization and reduced its bias toward one class, as indicated by more consistent loss and accuracy curves. However, given other imbalanced and medical situations, additional metrics are critical. These include precision, model predictions in a given class, and metrics such as recall, F1 score, and confusion matrices.

Below is a table (Table 3) for the evaluation result of the three models, EfficientNet-B0, ResNet50, and DenseNet201.

Observations:

1. The recall and F1 scores of EfficientNet-B0 improved significantly for Class 1 in the balanced setting for PEP prediction.
2. With augmentation, ResNet50 had the highest accuracy overall at 0.88, but had poor predictions for Class 1.
3. The recall and F1 scores of DenseNet201 were poor for Class 1 and, therefore, in this study, this model was the worst model for identifying PEP cases.
4. Despite applying balancing and augmentation strategies, several image-based models continued to show low sensitivity for Class 1, likely due to the limited number of positive PEP cases and the exploratory nature of the dataset.

### 4.4. Results of Multimodal Fusion

**Figure 16:**
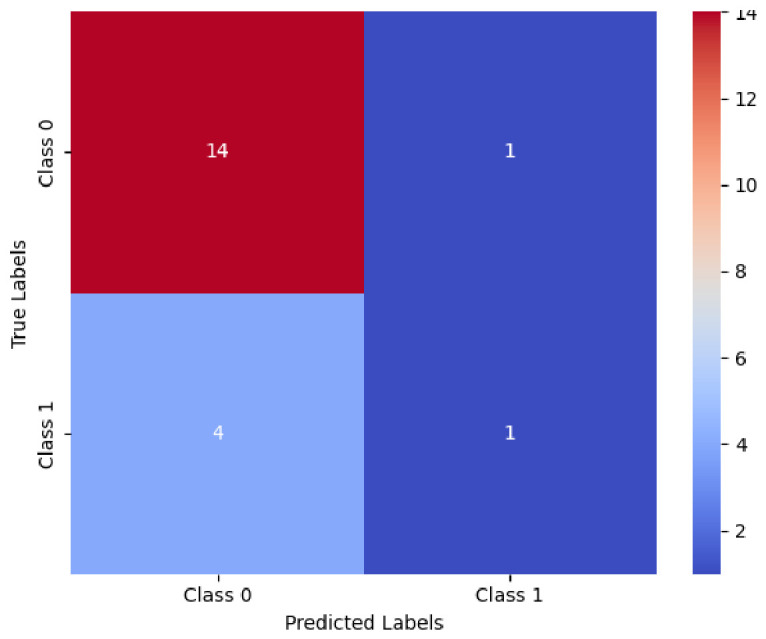
Multimodal Confusion Matrix.

A multimodal contrastive learning (MMCL)-based model was developed using a ResNet50 image encoder and a two-layer MLP-based tabular encoder. During self-supervised pretraining, image and tabular embeddings were aligned in a shared latent space using contrastive learning. During supervised fine-tuning, 2048-dimensional image and tabular features were concatenated to form a 4096-dimensional fused representation for binary PEP prediction. The model’s performance is summarized in terms of precision, recall, F1-score, and a confusion matrix, as shown in Table 4 and Figure 16.

#### Observations

1. The multimodal model demonstrated positive potential in identifying non-PEP cases (Class 0) with a recall of 0.93. The model correctly captured 14 of 15 non-PEP cases.
2. In contrast, positive PEP cases (Class 1) are more difficult to identify. For the 5 PEP cases, the model identified only 1. Thus, it has a Class 1 recall of 0.20.
3. This situation reflects the model’s surplus of specificity (classifying non-PEP cases), while the model also underperforms in sensitivity by failing to classify true PEP cases.
4. The reduced precision and F1 score for class 1 posited the fact that, in order to draw sufficient learning from a class, a sufficient quantity of minority cases was required for learning from the class.

## 5. Discussion

To evaluate the effectiveness of different learning approaches for predicting post-ERCP pancreatitis (PEP), we compared tabular, image-based, and multimodal fusion models. Class 1 represents patients who developed PEP, while Class 0 represents those who did not.

### 5.1. Comparison of Proposed Approaches

The performance comparison across tabular, image-based, and multimodal approaches highlights the relative strengths and weaknesses of each modality in classifying the target outcomes.

**Figure 17:**
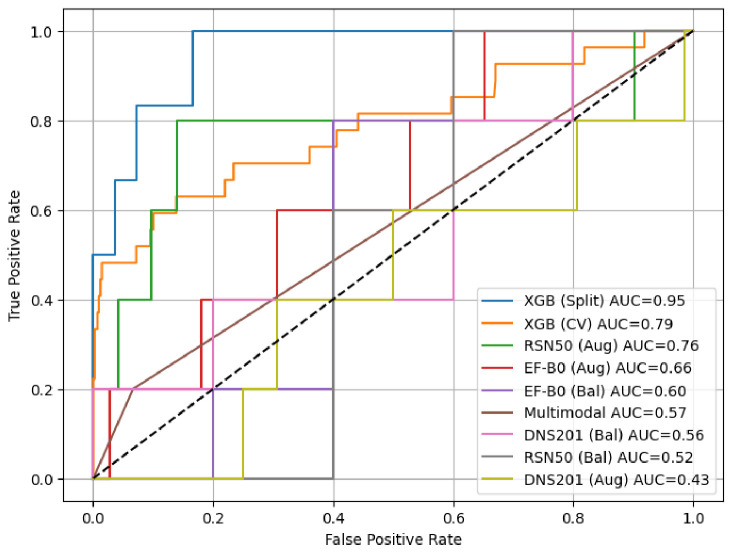
Overall ROC Curve Comparison. The black dashed line represents random performance (AUC = 0.5); curves above it indicate better performance.

The ROC curve Figure 17 shows that the XGBoost (train–test split) model performs best with an AUC of 0.95, followed by XGBoost (CV) at 0.79. Among image-based models, ResNet50 (Augmented) leads with an AUC of 0.76, while others, such as DenseNet201 (Augmented) perform poorly (0.43). The multimodal model achieves a moderate AUC of 0.57. Overall, tabular models outperform both image-based and multimodal approaches.

Observations:

Table 5 summarizes the following observations:

1. Tabular XGBoost models achieved the highest overall accuracy and the greatest performance and strongest F1’s across Class 1 and Class 2.
2. The image-based models (EfficientNet, ResNet50, DenseNet201) failed to detect Class 1 (PEP) unless the data was balanced, and DenseNet201 failed to detect Class 1 altogether in the augmentation function.
3. The dataset was improved through balancing to increase the recall and F1 for Class 1, especially with EfficientNet-B0 (F1 = 0.77).
4. The multimodal model has shown potential for integrating image data and tabular data, but additional tuning is necessary to increase the recall for Class 1 (PEP) cases.

Clinical Interpretation:

While tabular XGBoost achieved the highest overall accuracy and AUC, clinical performance is more dependent on sensitivity (recall) for Class 1 (PEP), as missing positive cases can have serious clinical consequences. Therefore, recall for Class 1 was a key evaluation metric in this study. Despite the application of balancing and augmentation strategies, several image-based and multimodal models continued to show limited recall for Class 1 (PEP), indicating difficulty in correctly identifying positive cases.

Although the multimodal framework successfully integrated endoscopic image and clinical data, it did not outperform the tabular models in the current study. One possible reason is the limited number of available image samples, as some procedural video data were unavailable or excluded due to data loss and quality limitations. In addition, deep learning-based image and multimodal models generally require larger datasets to learn robust feature representations compared with tabular machine learning models such as XGBoost. Therefore, the present study should be considered exploratory. Furthermore, the dataset was relatively small and collected from a single institute, which may limit generalizability. Despite the lower multimodal performance, the framework demonstrated the feasibility of integrating anatomical and clinical information. Future studies using larger multi-institutional multimodal datasets and improved image quality may further enhance predictive performance and generalizability.

### 5.2. Comparison with Prior Work (Based on Tabular AUC)

Table 6 compares the proposed XGBoost model with existing studies for post-ERCP pancreatitis (PEP) prediction using clinical tabular data. The proposed model, trained on 446 patients, achieved a cross-validation AUC of 0.786 and a test AUC of 0.95, outperforming previous studies that used larger datasets but reported lower AUC values (0.671–0.74). However, the model shows moderate sensitivity (recall = 0.48), indicating limited ability to detect all positive PEP cases. These results suggest that performance depends not only on dataset size but also on model design and preprocessing, and that further improvements are needed to enhance sensitivity and generalizability.

## 6. Conclusions

In this exploratory proof-of-concept study, we evaluated three approaches for predicting post-Endoscopic Retrograde Cholangiopancreatography (ERCP) pancreatitis (PEP): tabular clinical data, endoscopic image data, and multimodal fusion. Among the evaluated approaches, the tabular-based XGBoost model achieved better predictive performance; however, its performance should be interpreted cautiously due to the limited dataset size and the small number of positive PEP cases. The image-based and multimodal models demonstrated lower performance and only moderate sensitivity in detecting positive PEP cases. These findings suggest that, within this dataset, structured clinical variables currently provide stronger predictive signals for PEP than image-only or multimodal approaches. Nevertheless, the limited number of positive PEP cases and the single-center retrospective design reduced the stability and generalizability of the results, particularly for the image and multimodal-based models.

Future studies should prioritize larger multicenter datasets, sensitivity-focused optimization, advanced multimodal techniques, and generative AI augmentation [31,32] to improve positive case detection and robustness before clinical deployment.

## Figures and Tables

**Table 1:** Tabular Train and Test Split Classification Report.

	Precision	Recall	F1-Score	Support
0	0.97	1.00	0.98	84
1	1.00	0.50	0.67	6
accuracy			0.97	90
macro avg	0.98	0.75	0.82	90
weighted avg	0.97	0.97	0.96	90

**Table 2:** Cross Validation Classification Report.

	Precision	Recall	F1-Score	Support
0	0.97	0.97	0.97	419
1	0.52	0.48	0.50	27
accuracy			0.94	446
macro avg	0.74	0.73	0.73	446
weighted avg	0.94	0.94	0.94	446

**Table 3:** Image Data Transfer Learning Result Comparisons.

Approach	Model	Acc	Prec (C0)	Prec (C1)	Rec (C0)	Rec (C1)	F1 (C0)	F1 (C1)
Images Augmentation	Efficient B0	0.83	0.94	0.10	0.88	0.20	0.91	0.13
Image Balance		0.70	1.00	0.62	0.40	1.00	0.57	0.77
Images Augmentation	Resnet50	0.88	0.96	0.25	0.92	0.40	0.94	0.31
Image Balance		0.50	0.50	0.50	0.40	0.60	0.44	0.55
Images Augmentation	Densenet201	0.82	0.93	0.00	0.88	0.00	0.00	0.90
Image Balance		0.5	0.00	0.50	0.00	1.00	0.00	0.67

Acc = Accuracy; Prec = Precision; Rec = Recall; F1 = F1-Score. C0 = No PEP, C1 = Yes PEP. All metrics are computed separately for each class.

**Table 4:** Multimodal Classification Report.

	Precision	Recall	F1-Score	Support
Class0	0.78	0.93	0.85	15
Class1	0.50	0.20	0.29	5
Accuracy			0.75	20
Macro avg	0.64	0.57	0.57	20
Weighted avg	0.71	0.75	0.71	20

**Table 5:** Overall Result Comparison.

Approach	Model	Acc	Prec (C0)	Prec (C1)	Rec (C0)	Rec (C1)	F1 (C0)	F1 (C1)
Tabular	XGBoost	0.97	0.97	1.00	1.00	0.50	0.98	0.67
Tabular CV		0.94	0.97	0.52	0.97	0.48	0.97	0.50
Images Augmentation	Efficient B0	0.83	0.94	0.10	0.88	0.20	0.91	0.13
Image Balance		0.70	1.00	0.62	0.40	1.00	0.57	0.77
Images Augmentation	Resnet50	0.88	0.96	0.25	0.92	0.40	0.94	0.31
Image Balance		0.50	0.50	0.50	0.40	0.60	0.44	0.55
Images Augmentation	Densenet201	0.82	0.93	0.00	0.88	0.00	0.00	0.90
Image Balance		0.5	0.00	0.50	0.00	1.00	0.00	0.67
Mutimodal	Resnet50 + Multilayer Perceptron	0.75	0.78	0.50	0.93	0.20	0.85	0.29

Acc = Accuracy; Prec = Precision; Rec = Recall; F1 = F1-Score. C0 = No PEP, C1 = Yes PEP. All metrics are computed separately for each class. CV = Cross Validation.

**Table 6:** AUC-Based Evaluation of Proposed and Existing Tabular Models for PEP Risk.

Study	Year	Dataset Size	Model	Data Type	Recall (CV)	AUC (CV)	AUC (Test)
Proposed (This Study)	2026	446 patients (Tabular)	XGBoost	Clinical Tabular	0.48	0.786	0.95
Prior Study [30]	2026	1190 patients	CatBoost	Clinical Tabular	0.74	0.688	-
Prior Study [13]	2025	7389 patients	GBM	Clinical Tabular	0.65	0.70	0.74
Prior Study [4]	2023	1150 patients	Gradient Boosting	Clinical Tabular	-	0.70	0.671

## Data Availability

The datasets analyzed during the current study are not publicly available due to patient privacy and ethical restrictions.

## Abbreviations

The following abbreviations are used in this manuscript:

ERCP	Endoscopic Retrograde Cholangiopancreatography
PEP	Post ERCP Pancreatitis
CNN	Convolutional Neural Network
SHAP	SHapley Additive exPlanations
AUC	Area Under the Curve
SimCLR	Simple Framework for Contrastive Learning of Visual Representations
NT-Xent	Normalized Temperature-Scaled Cross-Entropy Loss
NPV	Negative Predictive Value
GBM	Gradient Boosting Machine
XGBoost	Extreme Gradient Boosting
MLP	Multilayer Perceptron
CLIP	Contrastive Learning Image Pertaining
SIAG	Sindh Institute of Advanced Endoscopy and Gastroenterology
MMCL	Multimodal Contrastive Learning
ROCs	Receiver Operating Characteristics
